# Supplementary material for: RNA-seq mixology: designing realistic control experiments to compare protocols and analysis methods
Source: Nucleic Acids Res. 2016 Nov 28;45(5):e30. doi: 10.1093/nar/gkw1063 (PMC5389713; doi:10.1093/nar/gkw1063)
Supplement: Supplementary Data [file gkw1063_supplementary_data.zip › nar-02003-met-k-2016-File014.pdf]

Supplementary Materials for "RNA-seq mixology: designing realistic control experiments to compare protocols and analysis methods"

Aliaksei Z. Holik, Charity W. Law, Ruijie Liu, Zeya Wang,
Wenyi Wang, Jaeil Ahn, Marie-Liesse Asselin-Labat,
Gordon K. Smyth and Matthew E. Ritchie

October 19, 2016

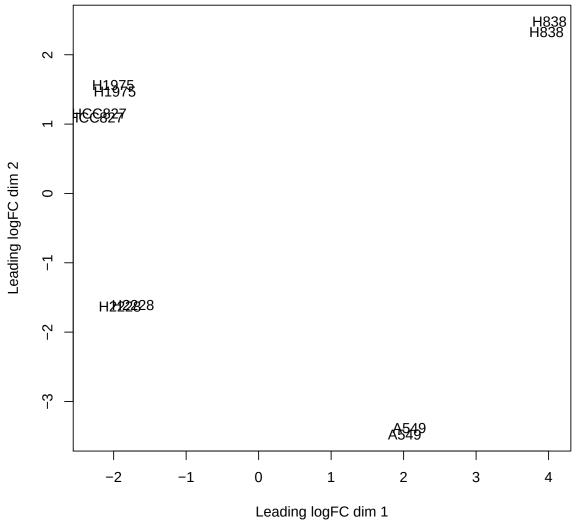

Supplementary Figure 1: Multidimensional scaling (MDS) plot for pilot RNA-seq experiment investigating gene expression in 5 lung adenocarcinoma cell lines (H2228, NCI-H1975, HCC827, H838 and A549). We chose the most similar two (HCC827 and H1975), which cluster together (top left of plot) in order to get more subtle fold-changes between samples that are more typical of what we might observe in a regular experiment.

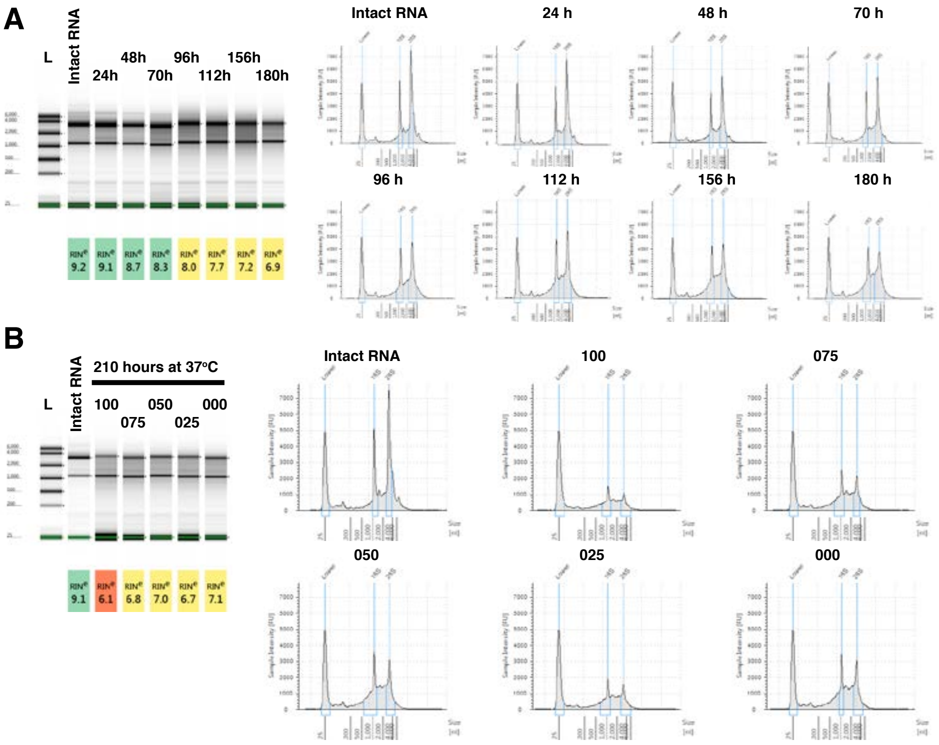

Supplementary Figure 2: Heat-mediated degradation of RNA samples. (A) An aliquot of RNA solution at approximately 100 ng/μl was incubated at 37°C and RNA integrity was assesses using RNA ScreenTape on the Tapestation instrument every 24 hours. RNA integrity number, which was used as an indicator of RNA quality, progressively decreased over the incubation period. (B) An aliquot from the second replicate of each mixture level was incubated at 37°C for approximately 9 days (210 hours). RNA quality of the degraded samples, as well as a representative sample of intact RNA, was profiled on RNA ScreenTape. RIN of the degraded samples was in the range between 6 and 7, compared to RIN 9 for the intact sample.

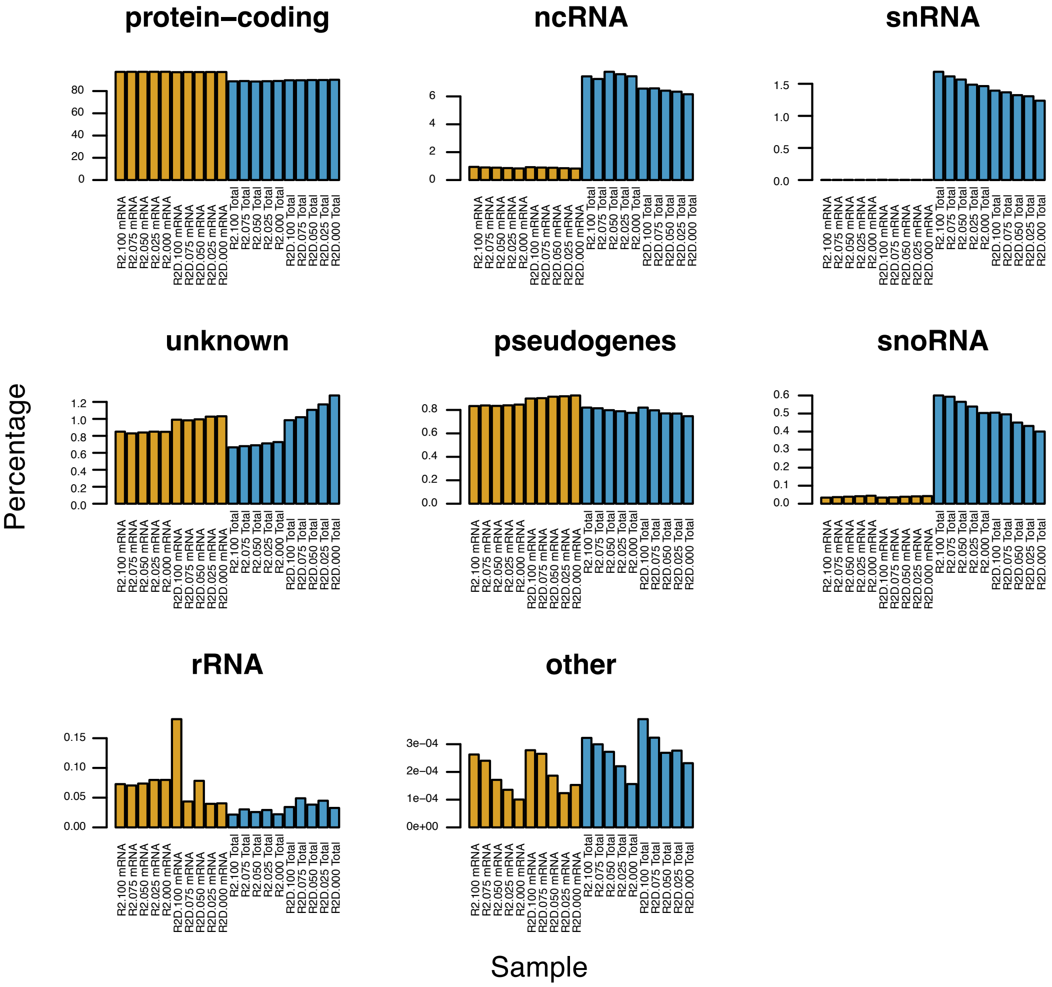

Supplementary Figure 3: Percentage of reads that map to different NCBI gene types for different library preparation methods (poly-A mRNA in orange and total RNA in blue) for the replicate 2 samples. The intact samples have labels that begin with *R2* and the degraded samples with *R2D*. The greatest differences between protocols are observed for non-coding RNAs (ncRNA), small nuclear RNAs (snRNAs) and small nucleolar RNAs (snoRNAs), with better recovery of each class from the total RNA protocol. The percentage of reads mapping to ribosomal RNAs (rRNAs) was lower for the total RNA protocol, indicating the effectiveness of the Ribozero depletion step.

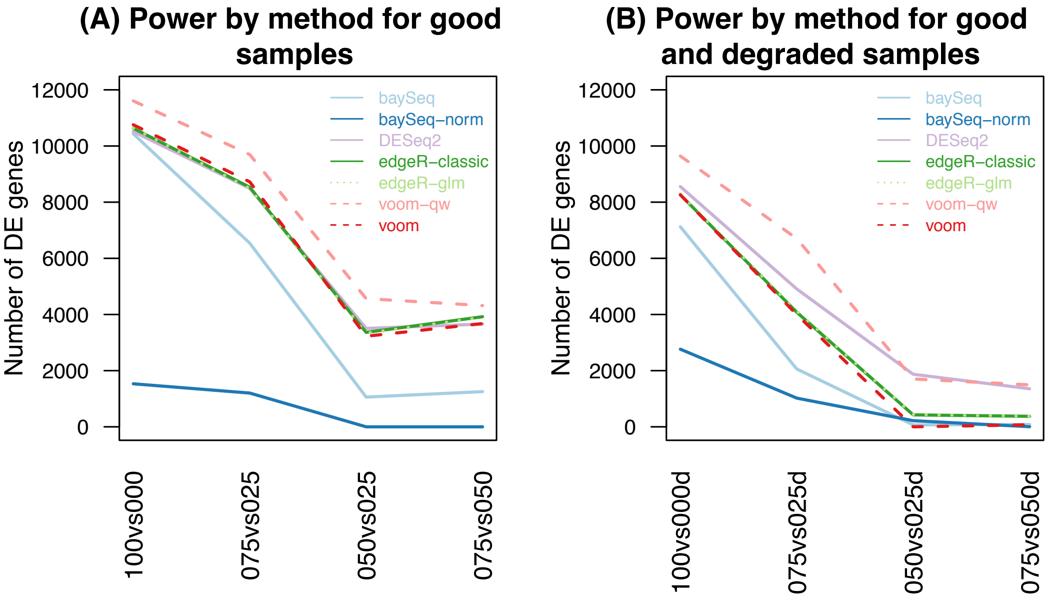

Supplementary Figure 4: Power for each method (y-axis) at a FDR cut-off of 0.05 using 'good' samples only (A) or good and degraded samples (B) for each contrast (x-axis) and a CPM cut-off of 1 in 3 or more samples to filter genes. Each method is depicted in a different colour and/or line type. The general trend for all methods is for the number of discoveries made to decrease as the RNA samples compared become more similar.

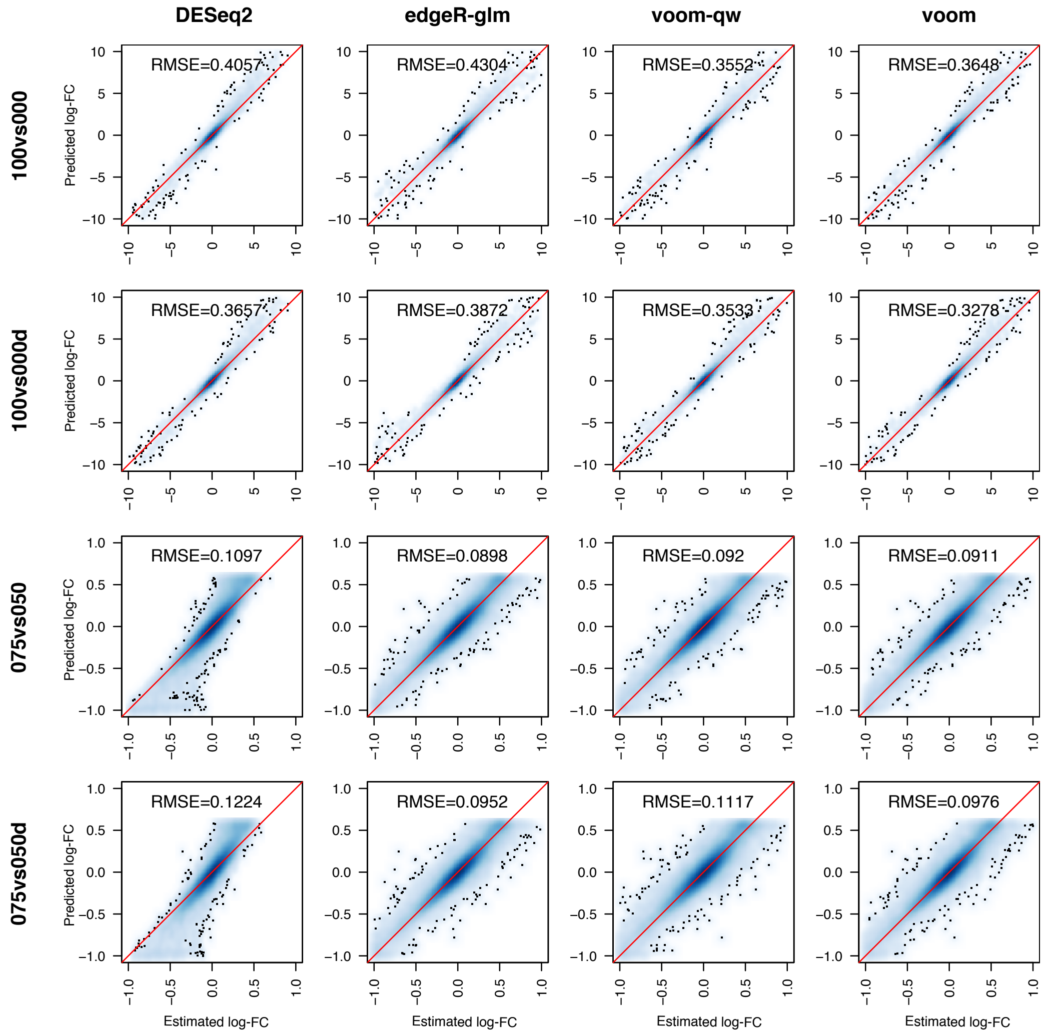

Supplementary Figure 5: Accuracy of log-FCs estimated by differential expression methods using CPM cut-off of 1 and using independent sample sets between the predicted and estimated log-fold changes. Out of a total of 20 samples, 6 samples (3 versus 3 comparisons) are used in each of the differential expression methods; the remaining 14 samples are used to calculate predicted log-fold changes using the nonlinear model (Equation 1). For example, in the 100vs000 comparison, a given method will use all of the good 100 and 000 samples to calculate log-fold changes; the corresponding predicted log-fold changes are then calculated for all 075, 050, 025 samples (good and degraded), plus the degraded 100 and 000 samples. The root-mean-square error (RMSE, Equation 3) between estimated and predicted log-FCs is displayed in each panel.

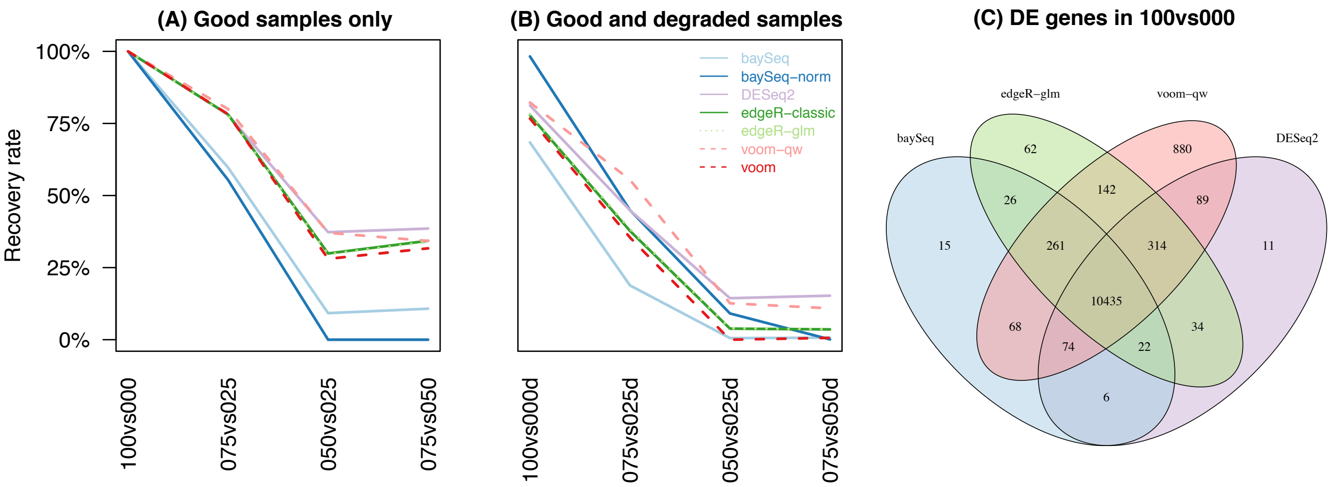

Supplementary Figure 6: Recovery rate of differential expression methods obtained using a CPM cut-off of 0.5 in 3 or more samples to filter genes. The rate at which DE genes are recovered from 100vs000 are displayed for comparisons using good samples only (A) and for those using good and degraded samples (B). Each method is shown in a distinct colour, with different line-types used when results are overlapping to allow them to be distinguished from one and other. The number of common DE genes for 100vs000 across select methods are displayed in (C). For a given software package, the method with higher recovery is shown, with exception to baySeq-norm due to its exceedingly high inconsistency rates.

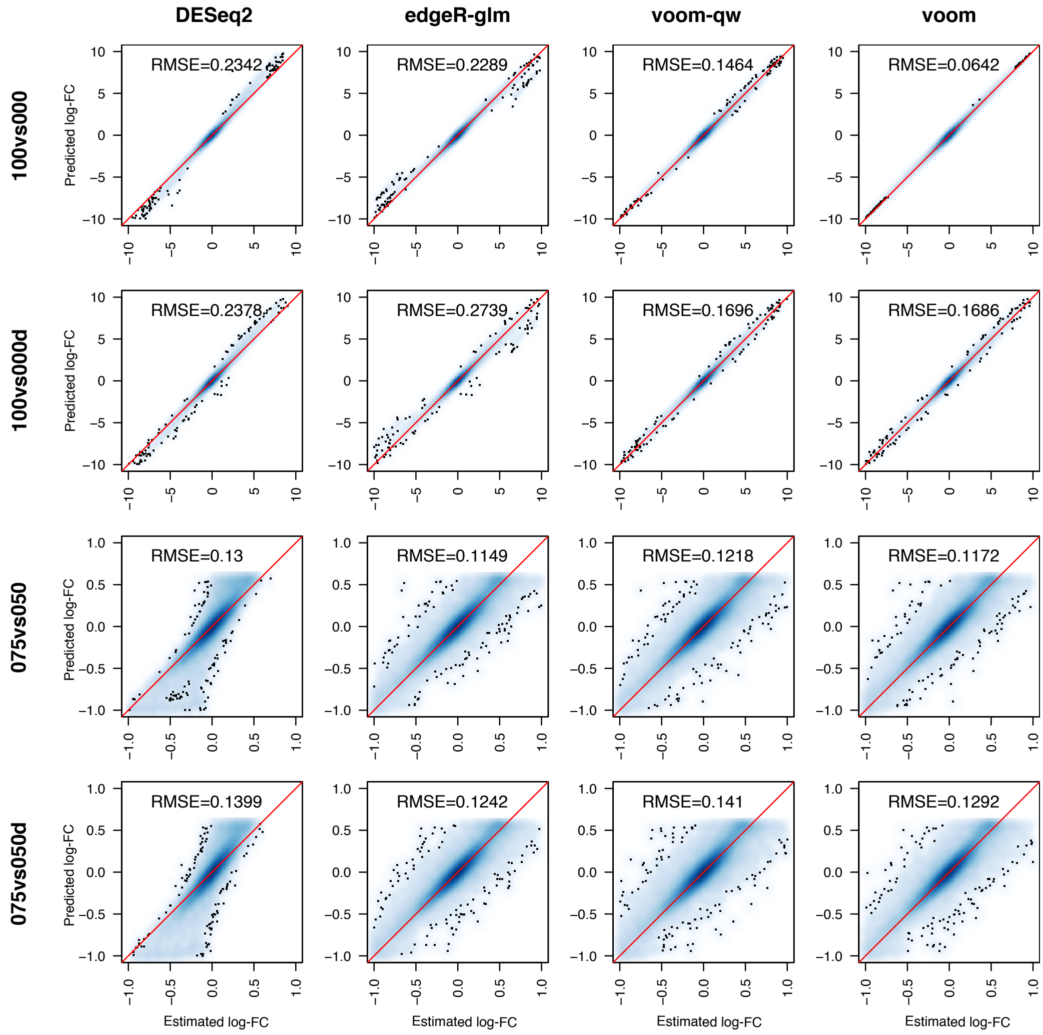

Supplementary Figure 7: Accuracy of log-FCs estimated by differential expression methods using CPM cut-off of 0.5 in 3 or more samples to filter genes. Gene-wise log-FCs estimated by the different methods are plotted on the x-axis against predicted log-FCs obtained from the nonlinear model on the y-axis. Areas with high density of points are shaded in blue, where colour intensity reflects the density of points. Red lines mark equality between estimated and predicted log-FCs. The root-mean-square error (RMSE, Equation 3) between estimated and predicted log-FCs is displayed in each panel.

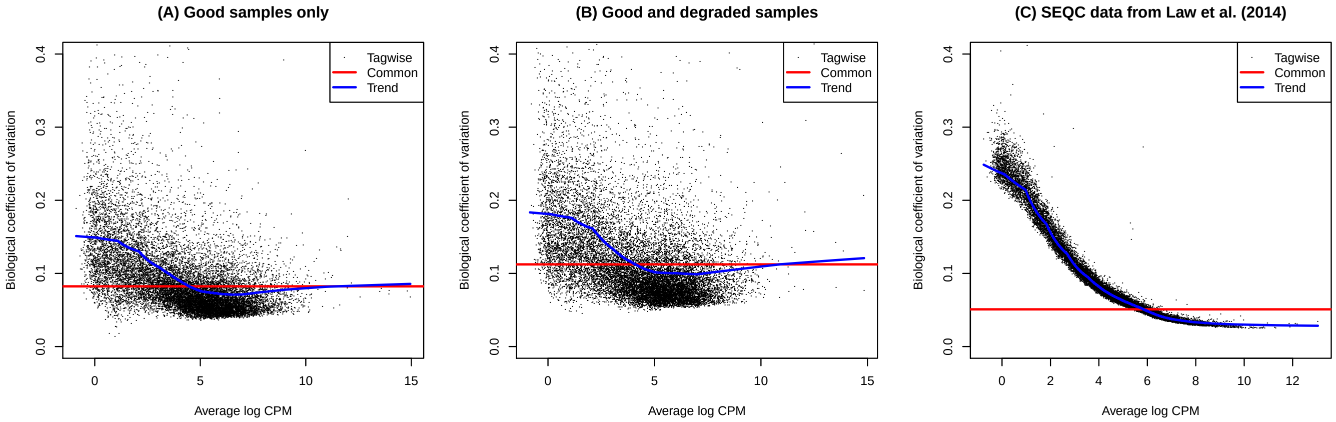

Supplementary Figure 8: Exploration of variability in the mixture experiment as measured by the biological coefficient of variation (BCV) from an *edgeR-glm* analysis fitted across the entire series using the good samples only (A) or the good and degraded samples (B). The inclusion of degraded samples sees the common BCV increases from 0.08 to 0.12. The Pilot SEQC data has the lowest common BCV of 0.05 (C). Gene-wise variability is also highly consistent in the SEQC dataset, with points very tightly distributed around the blue trend line (C), whereas in our mixture experiment (A-B), a considerable amount of vertical spread both above and below the trend line is observed. This indicates that there is far less biological variability in the SEQC data relative to our experiment.

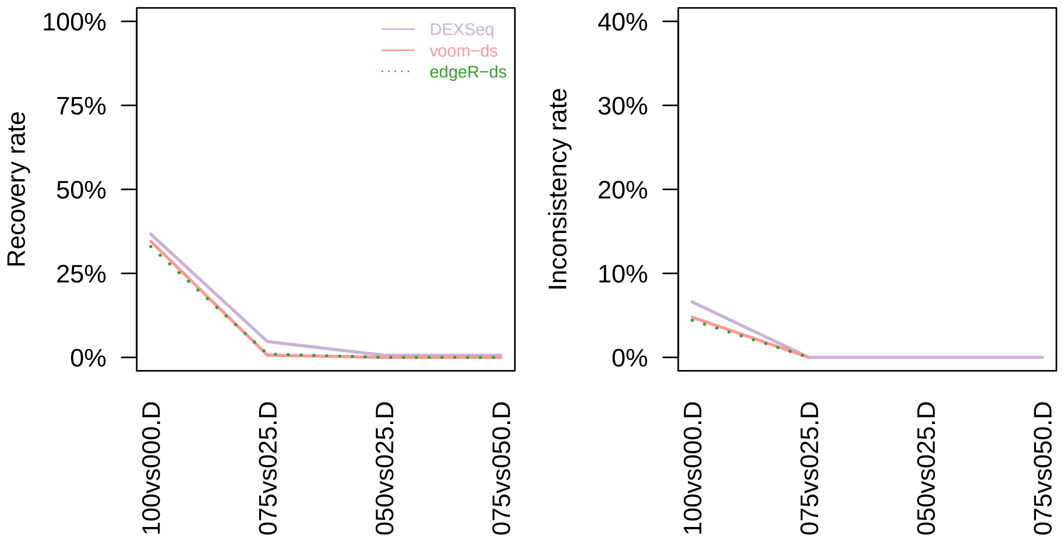

Supplementary Figure 9: Recovery and inconsistency rate of differential splicing methods using degraded samples. The rate at which DS genes are recovered from 100vs000 (A) and the inconsistency of detected genes (B) are displayed for comparisons using good and degraded samples. Each method is shown in a distinct colour, line-type combination.

Supplementary Table 1 Recovery values (number of genes and percentages) for each differential expression method in each comparison.

Contrast	*baySeq*	*baySeq-norm*	*DESeq2*	*edgeR-classic*	*edgeR-glm*	*voom-qw*	*voom*
100vs000	10435<br>100%	1532<br>100%	10513<br>100%	10631<br>100%	10641<br>100%	11606<br>100%	10757<br>100%
075vs025	6516<br>62.4%	942<br>61.5%	8413<br>80.0%	8452<br>79.5%	8420<br>79.1%	9532<br>82.0%	8611<br>80.0%
050vs025	1059<br>10.1%	0<br>0%	3504<br>33.0%	3362<br>31.6%	3334<br>31.3%	4553<br>39.0%	3225<br>30.0%
075vs050	1253<br>12.0%	0<br>0%	3657<br>35.0%	3915<br>36.8%	3900<br>36.7%	4313<br>37.0%	3671<br>34.1%
100vs000.D	7101<br>68.0%	1520<br>99.2%	8492<br>81.0%	8230<br>77.4%	8260<br>77.6%	9554<br>82.0%	8220<br>76.4%
075vs025.D	2059<br>19.7%	796<br>52.0%	4902<br>47.0%	4076<br>38.3%	4116<br>38.7%	6643<br>57.0%	3991<br>37.1%
050vs025.D	68<br>0.7%	181<br>11.8%	1871<br>18.0%	425<br>4.0%	436<br>4.1%	1703<br>15.0%	0<br>0.0%
075vs050.D	85<br>0.8%	3<br>0.2%	1356<br>13.0%	374<br>3.5%	379<br>3.6%	1493<br>13.0%	72<br>0.7%

Supplementary Table 2 Inconsistency values (number of genes and percentages) for each differential expression method in each comparison.

Contrast	*baySeq*	*baySeq-norm*	*DESeq2*	*edgeR-classic*	*edgeR-glm*	*voom-qw*	*voom*
100vs000	0<br>0.0%	0<br>0.0%	0<br>0.0%	0<br>0.0%	0<br>0.0%	0<br>0.0%	0<br>0.0%
075vs025	25<br>0.4%	260<br>22.0%	81<br>0.9%	81<br>0.9%	74<br>0.8%	165<br>1.7%	114<br>1.3%
050vs025	0<br>0.0%	0<br>0.0%	0<br>0.0%	1<br>0.0%	0<br>0.0%	12<br>0.2%	1<br>0.0%
075vs050	0<br>0.0%	0<br>0.0%	2<br>0.0%	5<br>0.1%	2<br>0.0%	5<br>0.1%	6<br>0.1%
100vs000.D	22<br>0.3%	1244<br>45.0%	63<br>0.7%	33<br>0.4%	33<br>0.4%	88<br>0.9%	41<br>0.5%
075vs025.D	0<br>0.0%	226<br>22.0%	7<br>0.1%	4<br>0.1%	4<br>0.1%	62<br>0.9%	5<br>0.1%
050vs025.D	0<br>0.0%	37<br>17.0%	0<br>0.0%	0<br>0.0%	0<br>0.0%	2<br>0.1%	0<br>0.0%
075vs050.D	0<br>0.0%	1<br>25.0%	0<br>0.0%	0<br>0.0%	0<br>0.0%	2<br>0.1%	0<br>0.0%
